# Do culturable seed endophyte communities differ between native and invasive Fabaceae sharing the same habitat?

**DOI:** 10.1111/plb.70120

**Published:** 2025-10-23

**Authors:** J. G. Jesus, F. Gonçalves, A. Clemente, H. Trindade

**Affiliations:** ^1^ Departamento de Biologia Vegetal, Faculdade de Ciências Universidade de Lisboa Lisbon Portugal; ^2^ CE3C—Centre for Ecology, Evolution and Environmental Changes & CHANGE—Global Change and Sustainability Institute Faculdade de Ciências da Universidade de Lisboa Lisbon Portugal

**Keywords:** *Acacia* spp., *Bacillus*, Dune, Forest, Microbial community, *Penicillium*

## Abstract

- Invasive plant species threaten ecosystems by decreasing biodiversity and altering their functioning. Recent findings suggest that endophytes play a crucial role in germination and early seedling development, which may enhance plant invasion success. This study aimed to characterize the culturable seed endophytes of invasive *Acacia* spp. and coexisting Portuguese native Fabaceae (*Erophaca baetica*, *Genista triacanthos*, *Retama monosperma*, *Stauracanthus genistoides* and *Ulex jussiaei*) within two habitats: Dune and Forest. We compared seed microbial endophytes, their richness, diversity, and functional traits.
- Microbial communities were obtained through classical microbiology, followed by 16S rRNA and ITS region sequencing for identification. The most relevant functional traits were predicted using FAPROTAX and FungalTraits.
- A total of 150 isolates, 99 bacterial and 51 fungal, were identified. A distinct clustering of microbial communities was observed in Dune and Forest, indicating environment dependency. Invasive *Acacia* spp. had the richer and more diverse culturable microbiomes when compared with native species, acquiring a subset of microbial partners shared with natives. In Dune habitat, there was higher similarity of seed endophyte communities between *Acacia* and native plants than in Forest. Functional traits were more diverse in invasive than in native species, especially for bacteria; fungi had functions complementary to bacteria in all plant species. General functions were related to metabolism, biocontrol and hormonal growth promotion, which are beneficial traits that enhance germination.
- This study highlights the ability of *Acacia* spp. to acquire locally beneficial endophytes in invaded areas, which may enhance their invasion success.

## Graphical abstract

Invasive *Acacia* seeds harbour richer and more functionally diverse endophyte communities than co‐occurring native Fabaceae, which could enhance establishment and invasion success of *Acacia*.

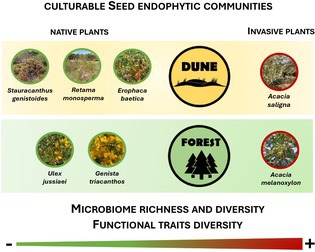

## INTRODUCTION

Biological invasions are one of the major threats to global plant conservation, often interacting synergistically with other threats, such as changes in land use, agriculture, biological resource use and climate change (Lughadha *et al*. 2020). The introduction of alien invasive plant species can have profound impacts on the structure and function of the ecosystems, by reducing plant diversity, competing for resources, and substantially altering the physical and/or chemical environment for the native species (Vilà *et al*. 2011; Simberloff *et al*. 2013; Kaul & Wilsey 2021). However, the severity of these effects is highly variable and depends on the invasiveness of the alien plant and the resistance and diversity of the native plant community (Levine & D'Antonio 1999; Ibáñez *et al*. 2021; Rilov *et al*. 2024).

Understanding the factors contributing to the success of plant invasions is crucial for predicting and managing the risk of new introductions and the spread of established invasive species. Previous studies aiming to identify the determinants of invasiveness have focused both on context‐dependent effects related to the introduction history, such as propagule pressure and residence time (Lockwood *et al*. 2005; Wilson *et al*. 2007), and on species phylogeny, biogeography and functional traits (Pyšek & Richardson 2006; van Kleunen *et al*. 2010; Gallagher *et al*. 2015).

Australian acacias have been introduced worldwide and several have become invasive, being responsible for reducing native species diversity (Marchante *et al*. 2003), changing nutrient cycles (Marchante *et al*. 2008; Ulm *et al*. 2017), water availability (Werner *et al*. 2010), and fire regimes (i.e. fire frequency and/or intensity) (Le Maitre *et al*. 2011). Considering these severe impacts, *Acacia* spp. serve as a model in invasion ecology studies (Richardson *et al*. 2023). Their invasiveness has been associated with a set of traits, including high growth rates and high resource use (Rodríguez‐Echeverría *et al*. 2009; Werner *et al*. 2010), massive flowering (Correia *et al*. 2014), generalist pollination systems, large and persistent soil seed banks and fire stimulated germination (Gibson *et al*. 2011; Le Maitre *et al*. 2011; Passos *et al*. 2017).

The ability of *Acacia* spp. to establish nitrogen‐fixing symbioses with bacteria enhances establishment and growth in diverse habitats, especially in nutrient‐poor soils (Rodríguez‐Echeverría *et al*. 2009) and in post‐fire regeneration (Jesus *et al*. 2020). Recently, attention has focused on plant–microbe interactions, including endophytes present in the seeds. This trait can play an important role in the invasion process, since seeds harbour diverse microbes important for plant health and productivity (Condessa *et al*. 2024).

In plant microbial assembly, small differences in early colonization processes may lead to large community differences during later life stages (Fukami 2015; Bergmann & Leveau 2022). Recent advances in Next‐Generation Sequencing techniques produce in‐depth knowledge of the microbial communities, increasing understanding of the set of microorganisms that might be involved in the invasion process (e.g. Simonin *et al*. 2022). However, these methods also have some limitations, and the use of classical microbiology allows establishment of a microbial collection, identified through sequencing methods, that can be explored later through functional and ecological perspectives.

Endophytes present in seeds can result from horizontal and vertical transmission, influenced by soil properties and environmental stresses, suggesting that their functional traits are relevant to overcome abiotic fluctuations (Truyens *et al*. 2013; Martiny *et al*. 2015). Also, endophytes can rapidly change their functional traits and, by being associated with plants, this adaptability is also transferable (Compant *et al*. 2010; Doty 2017). At the same time, the plant species selectively adopts different endophytes depending on its requirements (Garbeva *et al*. 2004; Wang & Zhang 2023). In addition, each plant generation incorporates a seed endophyte pool allowing their conservation and availability for the next generation (Johnston‐Monje *et al*. 2014), maintaining advantageous functional traits and discarding those that are no longer useful (Martiny *et al*. 2015). Although there is evidence of beneficial effects of seed endophytes on traits related to invasion (Molina‐Montenegro *et al*. 2023), the potential role of mutualistic seed endophytes in the invasion process has not been fully explored (Gioria *et al*. 2023). In invasive *Acacia* spp., seed traits have been identified as important factors for invasiveness, namely the ability to produce persistent seed banks, which enhances colonization and spread over time and space (Le Maitre *et al*. 2011). The role of seed endophytes, however, remains mostly unexplored, and further knowledge can provide clues to global understanding of invasive plant behaviour.

In this context, our main goal was to determine if the culturable endophytic community in the seeds of invasive species is acquired from the surrounding environment, ultimately providing a competitive advantage. For this, we analysed the composition of culturable seed endophytes of co‐occurring invasive and native Fabaceae species in two Mediterranean habitats invaded by *Acacia* spp. Specifically, we aimed to (1) assess whether the culturable seed endophytic pools differed between native and invasive species within the same habitat, and (2) evaluate the potential functions associated with these culturable endophytes.

## MATERIAL AND METHODS

### Plant species and habitats

Two natural habitats invaded by *Acacia* spp. were selected in western Portugal: coastal sand dunes and a mixed oak and pine forest; hereafter Dune and Forest, respectively. Site selection ensured that we collected seeds from both native and invasive species sharing the same habitat and during peak seed dispersal period. Dune and Forest study sites were in *Península de Setúbal* (*Mata dos Medos*, 38°35′ N 9°11′ W, and *Tróia*, 38°26′ N, 8°50′ W; 60 m a.s.l.) and *Serra de Sintra* (38°45′ N 9°25′ W, 156–217 m a.s.l.), respectively. The climate is Mediterranean, with a hot and dry summer; mean monthly temperature of the hottest (August) and coldest (January) months is 23.2 and 10.1°C in the Dune, and 19.6 and 10.4°C in the Forest, respectively. Mean annual rainfall ranges from 736 mm in Dune to 884 mm in the Forest (data from the closest weather stations; Domingos 2008; IPMA 2024).

Xerophytic shrub communities are the dominant vegetation type in the Dune, often in the understorey of open pine or eucalyptus forests. Dominant shrubs include *Stauracanthus genistoides* (Brot.) Samp., *Halimium calycinum* (L.) K. Koch, *Juniperus turbinata* subsp. *turbinata* Guss., and *Corema album* (L.) D.Don. Soils are dominated by Pleistocene to Miocene sand and sandstone deposits (Costa *et al*. 1998). The Serra de Sintra has a rich and diverse flora of both Mediterranean and Atlantic origin. The vegetation at the study site is a pine (*Pinus pinaster*), eucalypt (*Eucalyptus globulus*), and cork oak (*Quercus suber*) mixed forest with an understorey of gorse and heath communities. Dominant shrubs include *Arbutus unedo* L., *Calluna vulgaris* (L.) Hull, *Erica arborea* L., *Cistus salviifolius* L., *Genista triacanthos* Brot., *Q. lusitanica* Lam. and *Ulex jussiaei* Webb. The mountain range was built on intrusive rocks of the Late Cretaceous Sintra Igneous Complex, which intruded Mesozoic sedimentary host rocks. Due to differential erosion, granite syenite, gabbro and diorite are prevalent over the sedimentary host rocks (Kullberg & Kullberg 2020). Lithosols are the dominant soil type in the region.

Within each habitat, native species from the Fabaceae were selected among the most common shrubs that set seed by early summer. *Acacia* species have been introduced in Portugal and are listed as invasive (Decreto‐Lei no. 92/2019, Portuguese Ministry of the Environment, 2019). For this study, these species were selected based on high abundance and invasiveness. Plant species sampled and their characteristics are provided in Table 1. Invasive species, mainly *Acacia* species, are present in both Dune and Forest habitats; however, while thickets of *Acacia* (e.g. *Acacia saligna* (Labill.) H.L. Wendl and *A. longifolia* (Andrews) Willd.) are interspersed in the Dune, tall trees (*A. longifolia*, *A. saligna*, *A. dealbata* Link and *A. melanoxylon* R.Br.) are abundant and often form dense stands within the Forest. These *Acacia* spp. are listed as invasive species according to Portuguese Legislation (Decreto‐Lei no. 565/99, Portuguese Ministry of the Environment, 1999) and are among the most problematic plants. *A. melanoxylon* and *A. saligna* are classified as having Major and Minor impacts, respectively, according to EICAT (Kumschick & Jansen 2023).

**Table 1:** Characteristics of plant species in this study, including habitat category (Dune or Forest), status (invasive or native), life‐form and natural habitat, according to Flora‐On (2024).

habitat category	status	species	life form	habitat
Dune	Native	*Erophaca baetica* (L.) Boiss	Protohemicryptophyte	Understorey or edge of evergreen woodlands, shrublands and subnitrophilous grasslands
Forest	Native	*Genista triacanthos* Brot.	Chamaephyte, Phanerophyte	Clearings or understorey of oak woodlands on acid soils
Dune	Native	*Retama monosperma* (L.) Boiss	Phanerophyte	Secondary sand dunes; open shrublands or understorey of coastal pine forests on disturbed sandy soils
Dune	Native	*Stauracanthus genistoides* (Brot.) Samp.	Phanerophyte	Xerophytic shrublands in the understorey of pine or cork oak open forests, coastal cliffs, secondary and inland sand dunes, on acid and sandy substrates
Forest	Native	*Ulex jussiaei* (Webb.)	Chamaephyte, Phanerophyte	Understorey or forest edges and coastal cliffs on acid soils in high rainfall areas
Forest	Invasive	*Acacia melanoxylon* (R. Br.)	Phanerophyte	Forest edges; dense stands on acid soils
Dune	Invasive	*Acacia saligna* (Labill.) H.L. Wendl.	Phanerophyte	Coastal dunes; forms dense stands

### Seed collection and characterization

Ten to 40 plants per species (number depending on seed availability) were sampled for mature fruits at each site during the dispersal period, in June 2021, except for *A. saligna*, where fruits were only collected from eight plants. Permits for collection were issued by the National Conservation Authority (*Instituto da Conservação da Natureza e das Florestas, Licença* 947‐2021‐REC and 948‐2021‐REC). Plant material was transferred to the laboratory, and seeds removed from the fruits and other debris using sieves. Infested or damaged seeds were discarded. All potentially viable seeds were stored in paper bags at room conditions until used. Twenty randomly selected seeds from each species were weighed individually to determine mean seed weight.

### Seed surface disinfection and germination

Seeds from each species were sterilized by successive submersion in 70% ethanol for 1 min, 5% sodium hypochlorite (commercial bleach) for 6 min, five rinses in sterile distilled water, then transferred to boiled sterile distilled water. Individual seed scarification was then performed with a sterile scalpel blade to promote germination. Seeds were germinated on 1% agar, incubated for 2 days at room temperature in darkness, then placed in an incubator at 28°C under continuous light, for 1 to 2 weeks until seed germinated (i.e. radicle emergence). Plates were discarded if fungal or bacterial growth occurred during incubation.

### Isolation and characterization of culturable seed endophytic microbes

Approximately 3 g germinated seeds per species (varying from 1 seed of *Erophaca baetica* and *Retama monosperma*, to 30 seeds of *Genista triacanthos*) were macerated with a mortar and pestle with 1 mL distilled water then inoculated on Yeast Mannitol Agar (YMA) and Potato Dextrose Agar (PDA) (Thermo Fisher Scientific) plates at 28°C, for isolation of bacteria and fungi, respectively. New isolations were performed for each plant species until no new morphologically distinctive colonies were obtained.

Following bacteria and fungi isolation and purification, the axenic cultures were macroscopically examined and characterized. For bacteria, routine KOH, catalase and oxidase tests were performed, as described in Condessa *et al*. (2024). Also, a Gram‐stained slide was prepared for optical microscope observations. Phenotypic and biochemical characterization were considered for clustering analyses, together with molecular fingerprinting.

### DNA extraction and fingerprinting

Extraction of DNA followed a modified protocol with GES (guanidium thiocyanate, EDTA and Sarkosyl; Pitcher *et al*. 1989), with an incubation at a higher temperature (60°C instead of 37°C in the original protocol) and a single wash step with cold absolute ethanol. A loop of one pure colony was used as starting material, and the final resuspension was in 50 μL TE (Tris‐EDTA). DNA from all isolates was amplified through polymerase chain reaction (PCR) using the universal primers csM13 (5′‐GAGGGTGGCGGTTCT‐3′) (Meyer *et al*. 1993), (GTG)_5_ (5′‐GTGGTGGTGGTGGTG‐3′) (Versalovic *et al*. 1994) and PH (5′‐AAGGAGGTGATCCAGCCGCA‐3′). These primers are non‐specific and have been routinely used to amplify DNA from different species to allow fingerprinting. This involves comparison of DNA profiles and is a common procedure for preliminary study of microbial collections. The PCR mix contained 1 U Taq DNA polymerase (NZYTaq II, Nzytech), 3 mM MgCl_2_, 1× Reaction buffer, 0.2 mM of each dNTPs, 0.2 pmol μL^−1^ primer and 2 μL template DNA, in a final volume of 25 μL. The PCR was performed in a BioRad T100 thermal cycler (BioRad, USA), starting with an initial cycle of 5 min at 95°C, followed by 40 cycles of 1 min at 95°C, 2 min at 50°C and 2 min at 72°C, and a final extension for 5 min at 72°C. A 10% random replication was performed considering the total of isolates obtained from each plant species. The amplified PCR products were separated by electrophoresis for 5 h at 85 V on 1% (w/v) agarose gel in 0.5× TBE buffer. The gel was stained with 0.5 ng μL^−1^ ethidium bromide for 10 min, visualized under UV light in a transilluminator using the software Alliance 4.7 (Uvitec, Cambridge, UK) and processed with the associated software, Alliance v. 15.15 (Uvitec).

Comparison of DNA profiles of each isolate from each primer (csM13, GTG5, and PH) was performed using BioNumerics (Applied Maths N.V., 1998), following a cluster analysis based on the Pearson correlation coefficient and agglomerative hierarchical clustering Unweighted Pair‐Group Method with Arithmetic Mean Algorithm (UPGMA). Similarly, a composite cluster analysis was performed based on the three primers. The 10% replicated profiles allowed establishment of the cut‐off level of the dendrogram (Figs. S1 and S2).

### Taxonomic identification of bacterial and fungal endophytes via 16S and ITS rRNA sequencing

The 16S rRNA and ITS genes were amplified through PCR, followed by sequencing to identify the bacterial and fungal isolates, respectively. For 16S rRNA, amplification was performed using the primer combination PA(8f), (5′–AGAGTTTGATCCTGGCTCAG‐3′) (Massol‐Deya *et al*. 1995) with 907r (5‐CCGTCAATTCMTTTRAGTTT‐3), while for ITS, the primer pair ITS5 (5′‐GGAAGTAAAAGTCGTAACAAGG‐3′) and ITS4 (5′TCCTCCGCTTATTGATATGC‐3′) was used. Isolates representative of all clusters obtained after fingerprinting were included. The mix was similar to that used previously, except 2 mM MgCl_2_ was used. The PCR program started with an initial cycle of 3 min at 94°C, followed by 35 cycles of 1 min at 94°C, 1 min at 55°C, 1 min at 72°C, and a final extension for 3 min at 72°C. All PCR products were purified (according to STABVIDA routine procedures) and sequenced using the reverse primer (STAB VIDA, Costa da Caparica, Portugal).

The DNA sequences were edited and analysed using the software Geneious (v. 5.3 2010, https://www.geneious.com). All the sequences were compared with data available in GenBank using BLAST for 16S ribosomal RNA and ITS databases, for bacteria and fungi, respectively. Analysis of clusters on the dendrogram obtained following fingerprinting allowed us to identify isolates that were in the same cluster. All DNA sequences were submitted to GenBank (accession nos. PP703184–PP703237 for bacteria; PP704364–PP704397 for fungi; Tables S1 and S2).

### Diversity analysis and statistics

To explore differences in culturable microbial community composition among the studied plants within each habitat, a Principal Coordinates Analysis (PCoA) was performed using Bray–Curtis dissimilarity to construct the distance matrix, including all identified Operational Taxonomic Units (OTUs). Also, we performed non‐metric multidimensional scaling (NMDS) on the microbial communities obtained to explore relationships among plant endophyte communities of each habitat. Furthermore, endophytic microbial communities were plotted *per* plant species using cumulative relative abundance, excluding unidentified isolates. This lack of identification was considered when sequencing values were <90% or when identification was only possible above genus level.

The software BioNumerics (Applied Maths, Belgium) was used to construct dendrograms that compare and cluster DNA fingerprints. Shannon‐Weiner (H′) and Chao1 indices were calculated following Krebs (1989) to determine culturable microbial species diversity and richness in each plant species.

All isolated OTUs were considered for functional prediction based on FAPROTAX v. 1.2.6 (Louca *et al*. 2016) and FungalTraits (Põlme *et al*. 2020) for bacteria and fungi, respectively, at species level. Presence and absence matrices were built according to these databases and a cumulative relative abundance for each identified function for each plant species was calculated. The predicted functions ascribed to each isolated OTU are described in Tables S3 and S4.

Data analyses were performed using the package *stats*, *rstatix* and *microeco* in R (v. 4.2.2) (R Core Team 2022).

## RESULTS

Fabaceae varied in seed mass, ranging from 0.05 ± 0.02 g in *Genista triacanthos* to 3.58 ± 0.09 g in *Erophaca baetica*. Within habitats, *E. baetica* had the heaviest and *Stauracanthus genistoides* the lightest seeds (0.12 ± 0.02 g) in Dune, while in Forest corresponding species were *Acacia melanoxylon* (0.72 ± 0.08 g) and *G. triacanthos* (Table 2). Overall, seeds from species in Dune had a higher average mass compared to those in Forest.

**Table 2:** Seed mass (mean ± SE) of each studied plant species categorized by habitat (Dune or Forest) and status (Invasive or Native).

habitat	status	plant species	seed mass (g)	
Dune	Invasive	*Acacia saligna* (E)	0.46 ± 0.06	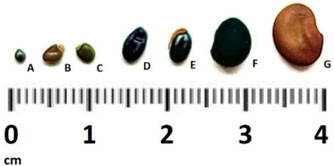
	Native	*Erophaca baetica* (G)	3.58 ± 0.09	
	Native	*Retama monosperma* (F)	3.00 ± 0.13	
	Native	*Stauracanthus genistoides* (C)	0.12 ± 0.02	
Forest	Invasive	*Acacia melanoxylon* (D)	0.72 ± 0.08	
	Native	*Genista triacanthos* (A)	0.05 ± 0.02	
	Native	*Ulex jussiaei* (B)	0.17 ± 0.02	

Letters in brackets correspond to labels on the right, which depict seeds of each species arranged by size.

Considering the culturable endophytic microbial pool (*viz* bacteria and fungi) isolated from seeds of each plant species, *E. baetica* had the highest number of OTUs (60), representing the highest value for both bacterial and fungal isolates, while *G. triacanthos* had the lowest, with only 9 isolates in total. Across all studied species, bacterial isolates were more abundant than fungal isolates, except in *R. monosperma* (Table 3). In Dune, *E. baetica* had the highest number of OTUs (22), followed by *A. saligna*, while in Forest, this highest value was for *A. melanoxylon* (39), which also had the highest number of isolates for both microbial groups.

**Table 3:** Total culturable bacterial and fungal operational taxonomic units (OTUs) isolated from seeds of each plant species categorized by habitat (Dune or Forest) and status (Invasive or Native).

habitat	status	plant species	bacterial isolates	fungal isolates	total
Dune	Invasive	*Acacia saligna*	19	4	23
	Native	*Erophaca baetica*	38	22	60
	Native	*Retama monosperma*	10	12	22
	Native	*Stauracanthus genistoides*	10	6	16
Forest	Invasive	*Acacia melanoxylon*	22	17	39
	Native	*Genista triacanthos*	5	4	9
	Native	*Ulex jussiaei*	16	3	19

**Fig. 1:**
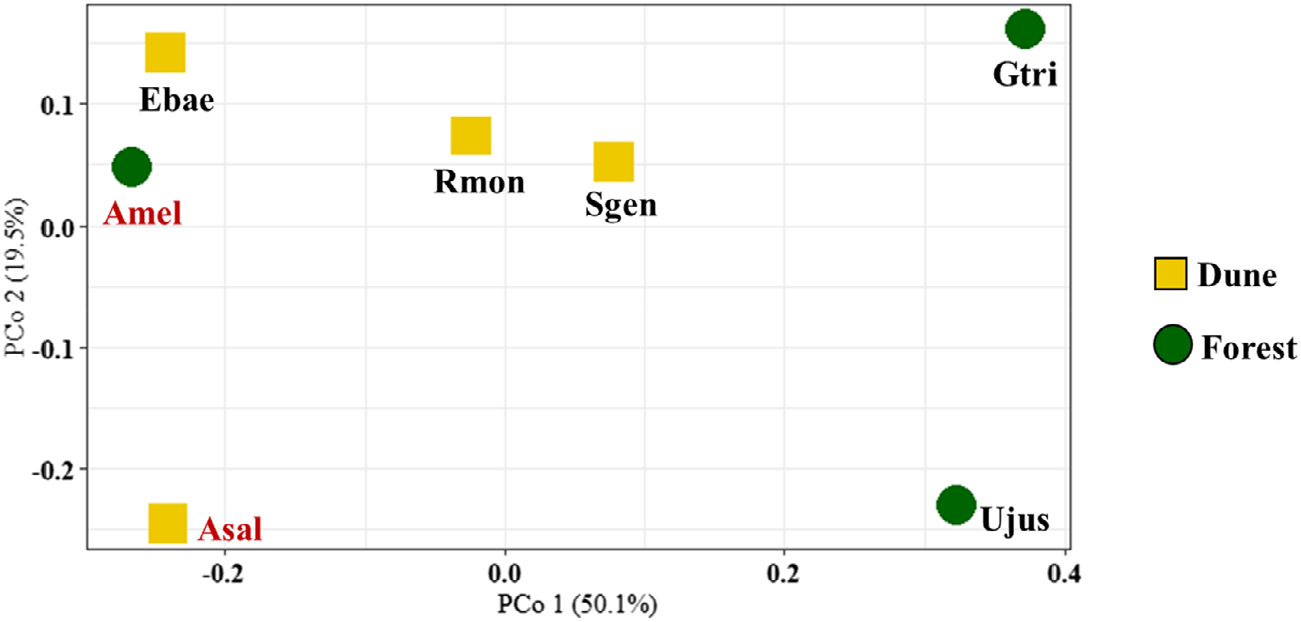
Principal coordinates analysis (PCoA) based on culturable, and classified genera of endophytic communities isolated from seeds of native and invasive plant species in Dune and Forest, using a Bray–Curtis dissimilarity matrix. Dune in yellow squares; Forest in green circles, while invasive species are in red. Plant species within habitats are represented accordingly [Invasive species: *Acacia melanoxylon* (Amel), *Acacia saligna* (Asal); Native species: *Erophaca baetica* (Ebae), *Genista triacanthos* (Gtri), *Retama monosperma* (Rmon), *Stauracanthus genistoides* (Sgen) and *Ulex jussiaei* (Ujus)].

The PCoA revealed higher dissimilarity among culturable endophytes from native species compared to invasives (Fig. 1) in Forest habitat, as also evidenced by NMDS (Fig. S3). There were more similarities among endophytic microbial communities in Dune.

**Fig. 2:**
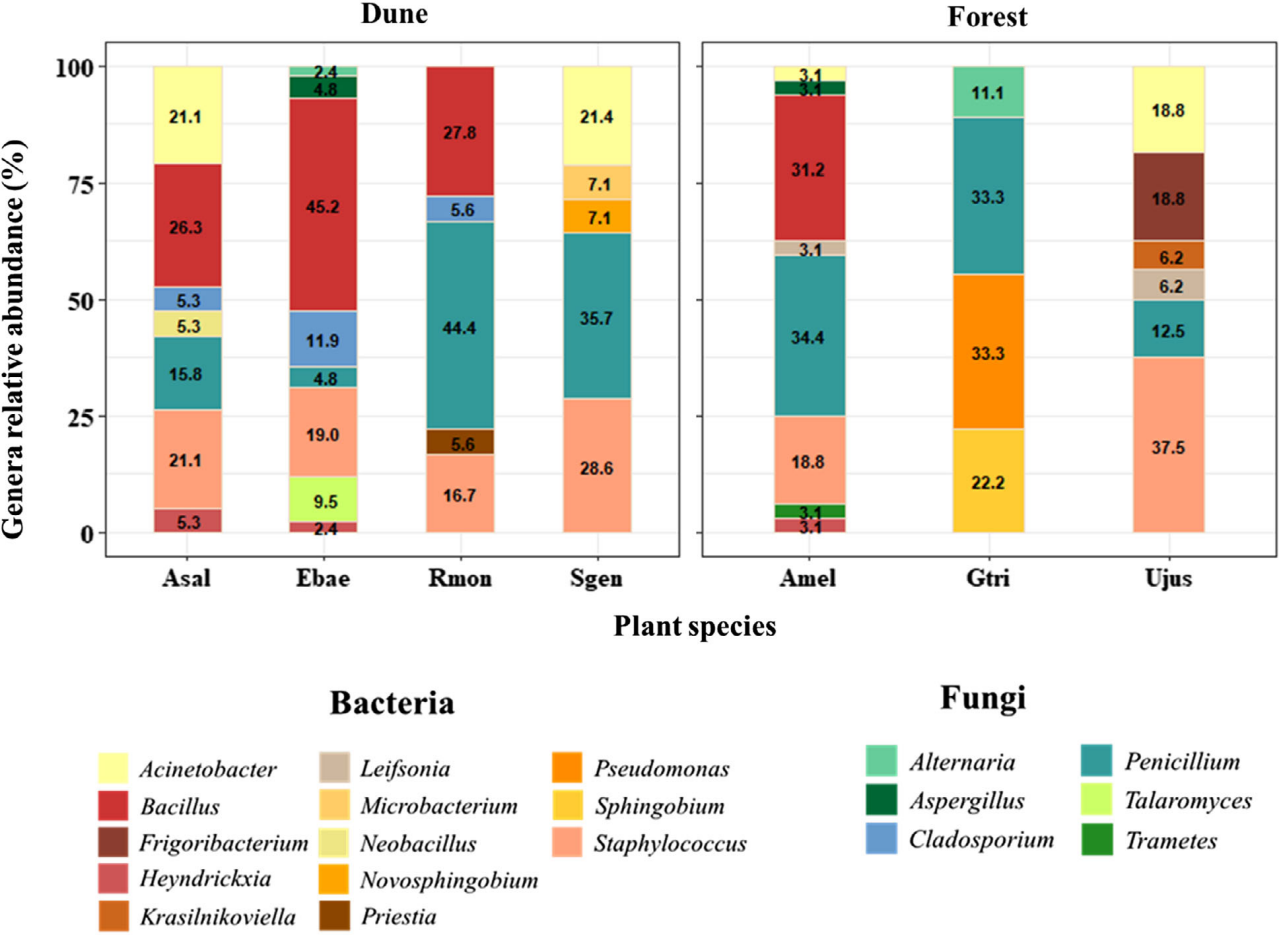
Seed endophyte pools (genus cumulative relative abundance) isolated from seeds of each plant species (plant species abbreviations as in Fig. 1) for each habitat (Dune or Forest). Colours are assigned to different microbial genera.

*Penicillium* was the only genus common to all plant species, despite differences in abundance: it was the most abundant microbe in *R. monosperma* (44.4%) and least abundant in *E. baetica* (4.8%; Fig. 2). For Dune, *Staphylococcus* was the only bacterial genus common to all plant species, while some microbial genera were exclusive: *Neobacillus* was only isolated from *A. saligna* (5.3%), *Talaromyces* from *E. baetica* (9.5%), *Priestia* from *R. monosperma* and *Microbacterium* from *S. genistoides* (7.1%). For Forest, *Trametes* was only found in *A. melanoxylon*, while *Alternaria*, *Sphingobium* and *Pseudomonas* were exclusive to *G. triacanthos. Frigobacterium* and *Krasilnikoviella* were only found in *U. jussiaei*. In general, Dune had more shared microbial genera among plant species (e.g. *Cladosporium* and *Bacillus*), while plant species from Forest had more distinct culturable microbiomes. *Acacia saligna* and *A. melanoxylon* had five genera in common (*Acinetobacter, Bacillus*, *Penicillium, Staphylococcus* and *Heyndrickxia*) and shared a culturable microbial community with coexisting natives, especially in Dune. *Acacia saligna* shared both bacterial and fungal endophytes with natives from Dune: *Heyndrickxia* with *E. baetica*, *Neobacillus* with *S. genistoides* and *Cladosporium* with both *E. baetica* and *R. monosperma*.

*Acacia* spp. had the highest richness (19 and 11 for *A. melanoxylon* and *A. saligna*, respectively), followed by the three native species from Dune (Table 4). *Genista triacanthos* and *U. jussiaei* had the lowest richness (4 and 1.79, respectively). Highest of diversity was among invasive species (0.97 and 0.92 for *A. melanoxylon* and *A. saligna*, respectively). Among native species, *G. triacanthos* had the lowest value (0.57).

**Table 4:** Richness (Chao1) and diversity (Shannon‐Wiener) indices for each culturable endophytic microbial pool isolated from seeds of plant species, categorized by habitat (Dune or Forest) and status (Invasive or Native).

habitat	status	plant species	richness	diversity
Dune	Invasive	*Acacia saligna*	11.0	0.92
	Native	*Erophaca baetica*	9.33	0.86
	Native	*Retama monosperma*	7.00	0.73
	Native	*Stauracanthus genistoides*	6.50	0.83
Forest	Invasive	*Acacia melanoxylon*	19.0	0.97
	Native	*Genista triacanthos*	4.00	0.57
	Native	*Ulex jussiaei*	1.79	0.80

**Fig. 3:**
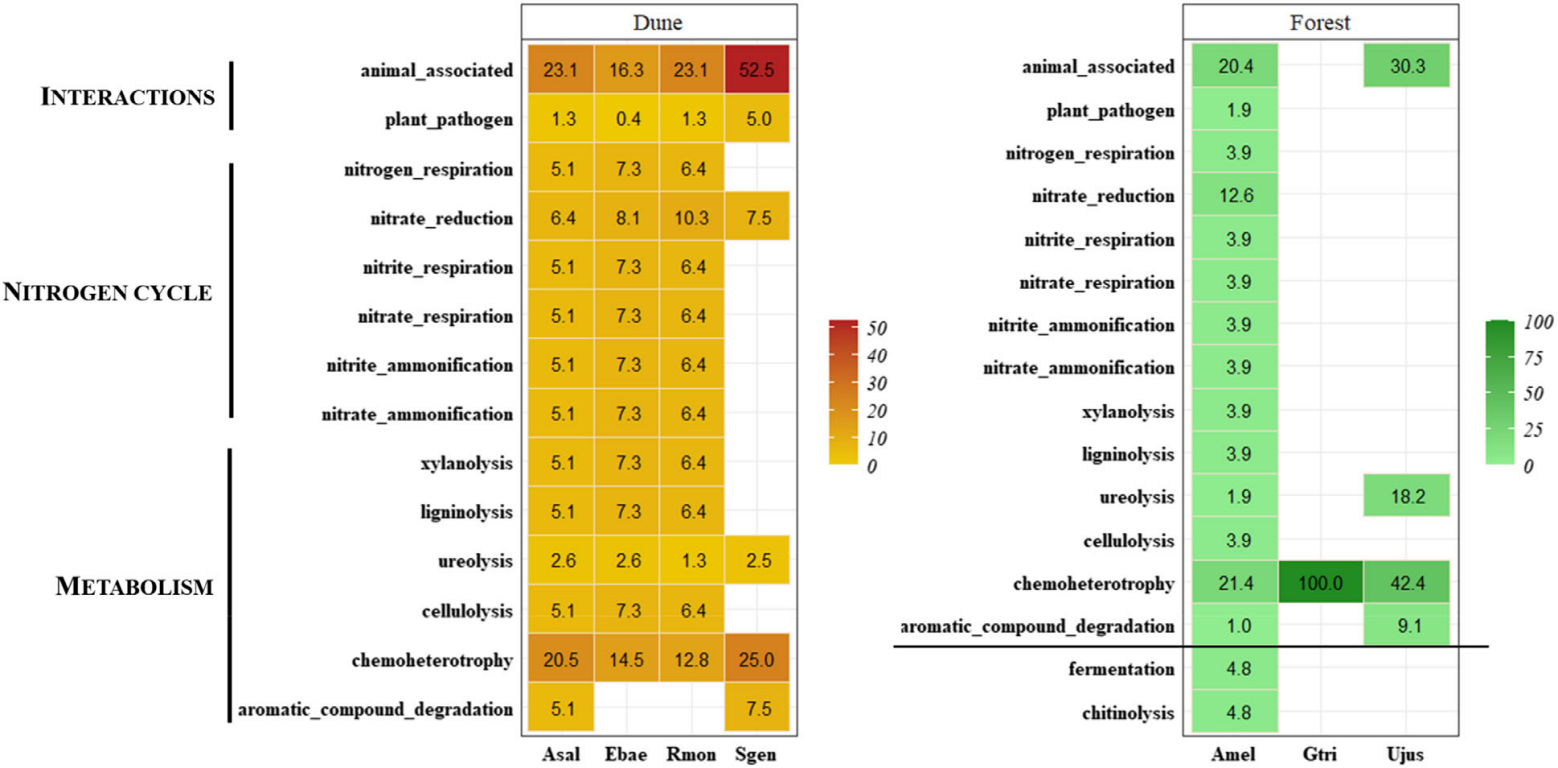
Bacterial functional prediction (cumulative relative abundance) at species level considering culturable microbial endophytes for each plant species [Invasive species: *Acacia melanoxylon* (Amel), *Acacia saligna* (Asal); Native species: *Erophaca baetica* (Ebae), *Genista triacanthos* (Gtri), *Retama monosperma* (Rmon), *Stauracanthus genistoides* (Sgen) and *Ulex jussiaei* (Ujus)] for each Habitat (Dune or Forest). Functional prediction was performed using FAPROTAX v1.2.6 (Louca *et al*. 2016).

Considering functional predictions of each bacterial OTU identified in each plant species, invasive *Acacia* spp. communities contained all the functions found within the respective habitat (Fig. 3). In Dune, functions were shared among culturable endophytes of both native and invasive plants. However, in Forest, *G. triacanthos* and *U. jussiaei* shared only one and four potential functions associated with their bacterial endophytes, respectively. *Ulex jussiaei* shared metabolism‐related functions or interaction with other organisms (i.e., animals) with the invasive *A. melanoxylon*. Fermentation and chitinolysis were unique to OTUs isolated from *A. melanoxylon* in Forest.

**Fig. 4:**
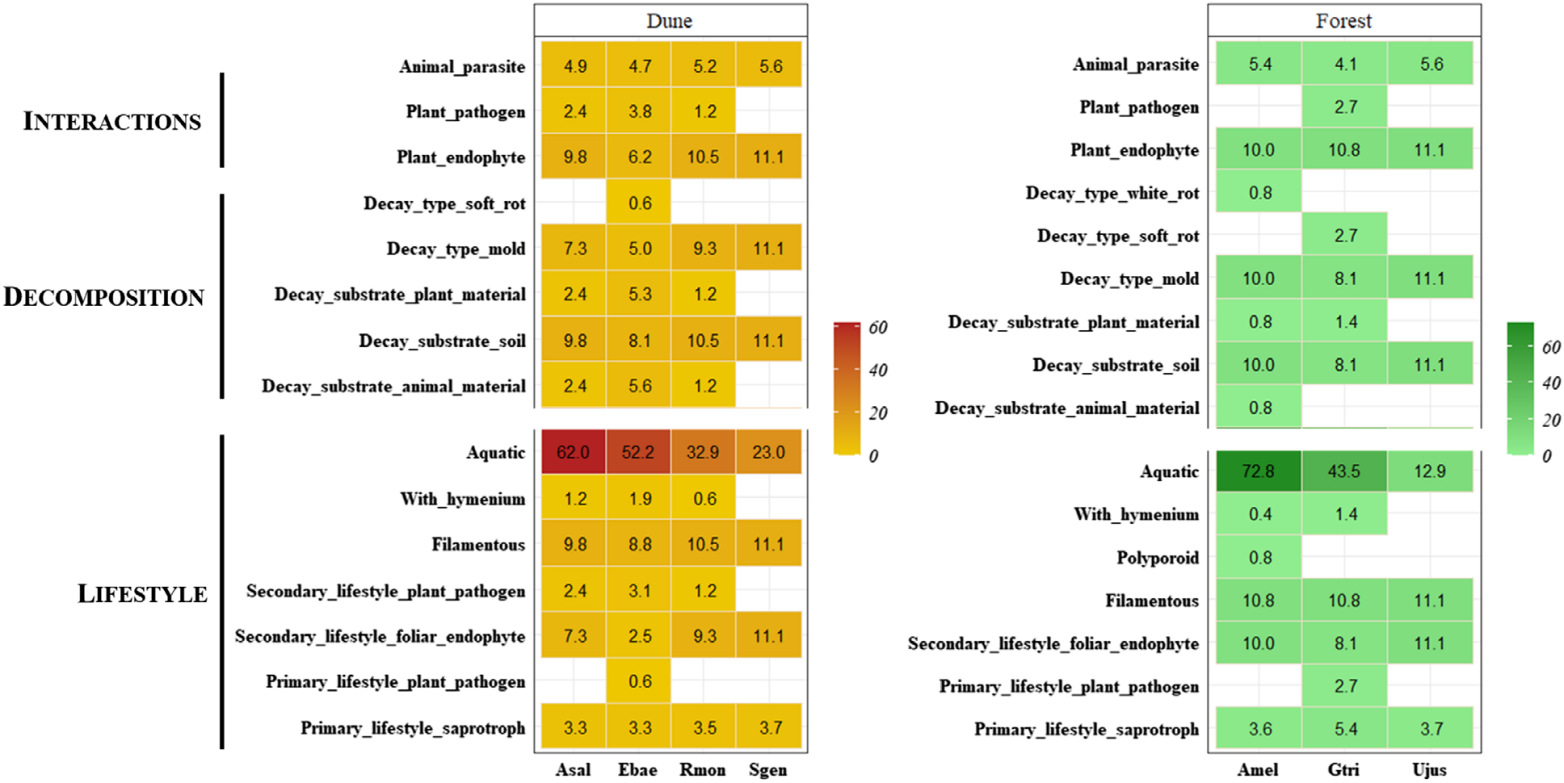
Fungal functional prediction (cumulative relative abundance) at species level considering culturable microbial endophytes for each plant species [Invasive species: *Acacia melanoxylon* (Amel), *Acacia saligna* (Asal); Native species: *Erophaca baetica* (Ebae), *Genista triacanthos* (Gtri), *Retama monosperma* (Rmon), *Stauracanthus genistoides* (Sgen) and *Ulex jussiaei* (Ujus)] for each habitat (Dune or Forest). Note that Interactions and Decomposition include functions, while Lifestyle include guils/traits. Functional prediction was performed using FungalTraits (Põlme *et al*. 2020).

In Dune, most predicted functions were common among all plant species, with culturable seed endophytes of *S. genistoides* having the lowest number of functions. The seed endophytes of *E. baetica* and *R. monosperma* shared all predicted functions and displayed similar relative abundance values. Among fungal functions and traits, aquatic origin was the only trait shared by all fungal endophytes across all plant species, while *E. baetica* had endophytes encompassing all identified functions and traits (Fig. 4). In Dune, fungal functions and traits were shared between invasive and native plant species, with similar abundance. Conversely, in Forest, potential functions were exclusive to either *A. melanoxylon* or *G. triacanthos*.

## DISCUSSION

In this study, the culturable, identified seed endophyte communities of coexisting native and invasive Fabaceae had similar compositions within each habitat, and dissimilarity when considering host status (invasive vs. native). Despite a well‐known cultivation bias, with the culturable microorganisms representing only a small proportion of the whole community (e.g. Sondo *et al*. 2023), patterns of higher diversity and richness were found in seeds of invasive plants compared to native seeds across both habitats. This culturable subset from the microbiome also had a broader range of potential functions, which might contribute to increased multifunctionality. Since many of these microbes were shared with native species from the same habitat, these findings could indicate the ability of *Acacia* to acquire beneficial microbes from co‐occurring native species (Martignoni & Kolodny 2024), but further research is needed to clarify the ecological significance of the differences and their contribution to invasion success. Also, plant species inhabiting Dune and Forest may differ in how they assemble microbial endophytes: the former relying more on environmental filters, and the latter on host selection (Bergmann & Leveau 2022; Jesus *et al*. 2025).

Research into microbial endophytes is increasing (White *et al*. 2019), given their role in enhancing plant resilience and response to abiotic stress (Wang & Zhang 2023). While much attention has been given to endophytes in plant organs, such as roots and nodules, seed endophytes are emerging as a key area (Raheem *et al*. 2018; Simonin *et al*. 2022). Studies on crops provide evidence that seed endophytes are beneficial to the plant (Fadiji & Babalola 2020; Maïwenn & Robin 2021). Seed fungal endophytes have recently been reported as an important factor explaining the invasiveness of *Poa annua* (Molina‐Montenegro *et al*. 2023). However, to the best of our knowledge, our study is one of the first to compare culturable seed endophyte communities between native and invasive plants coexisting in the same habitat.

The similarity in culturable taxa of the endophyte communities between *Acacia* and native species within habitats may reflect the capacity of *Acacia* to take advantage of local microbial resources during the invasion process. Acquisition of endophytes likely arises from horizontal transfer, as microbial endophytes with the most beneficial functional traits are frequently shared among different plants within the same community (Bright & Bulgheresi 2010). Although fungal endophytes are more host‐dependent (U'Ren *et al*. 2012; Wehner *et al*. 2014; Geisen *et al*. 2017), the community of bacterial endophytes from specific plant species is dependent not only on the host plant (Simonin *et al*. 2022) but also on the environment (Garbeva *et al*. 2004; Long *et al*. 2010). Therefore, plants growing in the same area tend to share common endophytes as they obtain them from the same “soil marketplace” (Johnston‐Monje *et al*. 2014). As *Acacia* spp. have long invaded the studied areas, they might have adopted seed endophytes common to native plants during an early stage of invasion. This opportunistic strategy might have enhanced their adaptive capacity, facilitating successful establishment and persistence in novel environments.

In this study, invasive Acacias had the highest richness and diversity of seed microbial endophyte communities, in comparison with native species. A previous study on the rhizosphere of plant invaders proposed an important role of microbiota in invasive plants success (Coats & Rumpho 2014 and references within). Here, we suggest the ability of Acacias to take advantage of local microbial sources that are shared with coexisting native species, as previously proposed by Martignoni & Kolodny (2024). Furthermore, our hypothesis aligns with Condessa *et al*. (2024), who observed differences in seed endophyte communities of *Acacia longifolia* between its native and invaded ranges, with some microbial taxa being exclusive to the invaded range. It is also relevant to consider the potential co‐introduction of microbes *via* Acacia seeds. Similarly to root‐nodules, compatible bacteria may be introduced during invasion, potentially disrupting native mutualisms (Rodríguez‐Echeverría *et al*. 2011, 2012) or fostering novel associations (Jesus *et al*. 2023). Introduced *Acacia* spp. may carry microbial endophytes in their seeds that promote establishment, which could then become available to native plant species.

All the microbial genera isolated from the plant species studied here have previously been identified as seed endophytes in other plant taxa (e.g. Samreen *et al*. 2021; Fagorzi & Mengoni 2022). Our study found that *Bacillus* as the most frequent genus identified in seeds, followed by *Staphylococcus*, which was present in all species except *G. triacanthos*. While several studies have documented the endophyte pool of some Fabaceae genera, they focused on different plant organs. For example, *Pseudomonas* spp. and *Bacillus* spp. were identified in nodules from *Genista* spp. (Dekak *et al*. 2020) and *Retama* spp. (Dahmani *et al*. 2020), respectively, and *Actinobacter* spp. were found in roots of *Ulex* spp. (Herrera *et al*. 2022). Within the genus *Acacia*, our research group previously characterized the seed bacterial endophyte community in *A. longifolia*, which included some of the endophyte species identified in the present study for *Acacia* species, namely *Staphylococcus* spp., *Bacillus* spp. and *Neobacillus* spp. (Condessa *et al*. 2024). The seed endophytes also included six fungal genera, with the most common belonged to the Ascomycota (Arnold *et al*. 2000; Wehner *et al*. 2014; Glynou *et al*. 2016; Geisen *et al*. 2017), especially *Penicillium* and *Cladosporium* (e.g., Rashmi *et al*. 2019). In the present study, *Penicillium* was the only fungal endophyte shared by all plant species, while *Cladosporium* was shared among species in Dune.

*Bacillus* and *Pseudomonas* spp. play important roles in nitrogen (N) fixation and phosphorus (P) and potassium (K) solubilization (Etesami *et al*. 2017; Velázquez *et al*. 2017). These are essential nutrients for plants, which explains their shared presence among plant species. *Staphylococcus* promotes plant growth by producing volatile organic compounds (Bitas *et al*. 2013). It is important to highlight that the functional traits identified in fungal endophytes complement those identified in bacterial endophytes in all plant species, making the seed a source of potential multifunctionality relevant for germination and early establishment, especially in dispersal events of invasive species and in post‐fire colonization.

Forest encompassed more plant‐specific microbial communities, reflected in more distinctive functionality among fungal endophytes when comparing invasive and native plant species. *Penicillium* is reported to enhance plant capacity to cope with abiotic stresses (Khan & Lee 2013), including salt stress, and promote resistance to pathogens (as revised by Wang & Zhang 2023). *Cladosporium* is a common genus contributing to the above‐mentioned NPK solubilization (Newcombe *et al*. 2022; Simonin *et al*. 2022), reinforcing its presence among endophytes of plant species, particularly in Dune.

All potential functions assigned to the different genera identified in the endophyte pools were present in *Acacia* spp. Functions related to metabolism, biocontrol and hormonal growth promotion enhance germination (Morales‐Cedeño *et al*. 2021; Lacava *et al*. 2022). Additionally, low P availability in the soil is a major factor limiting plant growth (Ågren *et al*. 2012), but all plant species studied contain seed endophytes that promote P availability, possibly by decomposing organic compounds in the soil. Moreover, our study revealed that plants from dry, nutrient‐poor and salt‐stress environments (Dune) share endophytes involved in ureolysis, a process crucial for soil enrichment through the degradation of organic N (Hasan 2000). The endophytic communities in Dune plants had similar potential functionality, likely reflecting high specialization and adaptation to harsh conditions. In contrast, Forest plants had distinct seed endophytic communities, with some genera exclusive to a specific host, which suggests host filtering. The invasive *A. melanoxylon* had a richer and more diverse endophytic community, while native plants tend to have fewer endophytes with more specific functions.

Our study suggests that the environment plays a role in modulating endophytes retained in seeds, making them available to the seedling. However, the host is known to have a significant role in selecting the microbial endophyte pool (Simonin *et al*. 2022). Both environment and host filters were identified as crucial to the assembly of microbial endophytes (Bergmann & Leveau 2022), extending the effects to a community scale. Endophytic communities are known to vary with habitat (Lau & Funk 2023), reinforcing the role of surrounding conditions in shaping microbial community composition. Our findings suggest that both host identity and environment contribute to assembly of seed endophyte communities. Specifically, we found increased similarity between endophyte communities of *Acacia* and those of native plants in the Dune habitat, where environmental filtering may have a stronger role. In contrast, communities in Forest appeared to be more host specific. These patterns align with studies showing that microbial community assembly is shaped by both host and environmental filters (Bergmann & Leveau 2022; Simonin *et al*. 2022), and that endophyte communities vary by habitat (Lau & Funk 2023). By comparing invasive and native Fabaceae species that coexist within the same environments, our study highlights the local imprint on seed microbiomes and supports the idea that seeds act as a reservoir of potentially multifunctional microbes. Although this study was based on a culturable collection of 150 isolates, these represent an important subset of the total seed endophyte communities. However, we acknowledge that the sample size constrains any broader generalization of our findings. Future studies should build on these results by expanding the sampling effort and employing high throughput tools, such as NGS, to more comprehensively characterize the seed endophyte community of the studied species and habitats.

Plant–microbe associations with generalist microbial communities have been proposed as a factor facilitating invasion success (Lau & Funk 2023). However, because the invasion process is context‐dependent and multiple sets of traits and factors might promote invasiveness in different environments, understanding the contribution of seed endophyte communities requires broader geographic scales. Furthermore, considering (1) fire stimulates seed germination in many *Acacia* spp., leading to population increases and often monospecific stands, and (2) fire has negative effect on soil microbiology, studies of seed endophyte communities in a post‐fire environment are essential.

## CONCLUSION

Our study identified 150 fungal and bacterial isolates, representing a relevant subset of the total seed endophyte community, including microbes potentially involved in seed and/or plant development. Invasive *Acacia* species have a more diverse culturable seed microbiome than co‐occurring native Fabaceae, with a broader range of potential functional traits. Many of these microbes are found in native species, suggesting that *Acacia* may establish associations with locally available microbial partners. Although the ecological roles of these microbes remain to be resolved, their presence may reflect *Acacia's* plasticity and opportunistic behaviour, potentially supporting its establishment and invasion success. The high similarity in endophyte communities among coexisting species in the Dune habitat, compared to the Forest, suggests that the relative influence of host and environmental filtering vary across habitats. While seed endophytes are only one of several factors contributing to invasion dynamics, further studies should explore their ecological roles, including comparisons between congeneric invasive and non‐invasive plants.

## AUTHOR CONTRIBUTIONS

MG led the data collection and curation. JGJ led the formal analysis, visualization and writing – original draft. AC and HT were responsible for supervision, validation and writing – review & editing. HT was involved in funding acquisition. All authors were responsible for conceptualization and gave final approval for publication.

## CONFLICT OF INTEREST

On behalf of all authors, the corresponding author confirms that there are no conflicts of interest.

## Supporting information


**Fig. S1.** Dendrograms of bacterial isolates for each plant species based on DNA fingerprinting: (a) *Acacia* melanoxylon, (b) *Acacia saligna*, (c) *Erophaca baetica*, (d) *Retama monosperma*, (e) *Stauracanthus genistoides* and (f) *Ulex jussiaei*. Identification based on 16S rRNA sequencing.
**Fig. S2.** Dendrograms of fungal isolates for each plant species based on DNA fingerprinting: (a) *Acacia melanoxylon*, (b) *Acacia saligna*, (c) *Erophaca baetica*, (d) *Retama monosperma*, (e) *Stauracanthus genistoides* and (f) *Ulex jussiaei*. Identification results based on ITS rRNA sequencing.
**Fig. S3.** Non‐metric Multi‐Dimensional Scaling (NMDS) based on culturable and classified genera of endophyte communities isolated from seeds of native and invasive plant species in Dune and Forest (stress value of 0.05). Ellipses represent the two habitats (Dune in yellow or Forest in green) and coloured squares and circles represent plant species within habitats [Invasive species: *Acacia melanoxylon* (Amel), *Acacia saligna* (Asal); Native species: *Erophaca baetica* (Ebae), *Genista triacanthos* (Gtri), *Retama monosperma* (Rmon), *Stauracanthus genistoides* (Sgen) and *Ulex jussiaei* (Ujus)]. Relative abundance of OTUs in each species was considered for ordination.
**Table S1.** BLAST analysis of sequences obtained from seed bacterial isolation from each plant species. The description shown is for higher percentage pairwise identity.
**Table S2.** BLAST analysis of sequences from fungal isolates from seeds of each plant species. The description shown is for higher percentage pairwise identity.
**Table S3.** Predicted functions for bacterial operational taxonomic units (OTUs), including number of OTU present in each plant species. Functions were predicted through bioinformatic tools using FAPROTAX v. 1.2.6 (Louca *et al*. 2016).
**Table S4.** Predicted functions for fungal operational taxonomic units (OTUs), including number of OTU present in each plant species. Functions were predicted through bioinformatic tools using FungalTraits (Põlme *et al*. 2020).

## Data Availability

Further data and details are provided in the Supplementary Information. The full dataset used in this study is available upon request to the corresponding author.
