# Supplementary material for: Do culturable seed endophyte communities differ between native and invasive Fabaceae sharing the same habitat?
Source: Plant Biol (Stuttg). 2025 Oct 23;28(1):249–60. doi: 10.1111/plb.70120 (PMC12710845; doi:10.1111/plb.70120)
Supplement: Supplementary file 1 — Fig. S1. Dendrograms of bacterial isolates for each plant species based on DNA fingerprinting: (a) Acacia melanoxylon, (b) Acacia saligna, (c) Erophaca baetica, (d) Retama monosperma, (e) Stauracanthus genistoides and (f) Ulex jussiaei. Identification based on 16S rRNA sequencing. Fig. S2. Dendrograms of fungal isolates for each plant species based on DNA fingerprinting: (a) Acacia melanoxylon, (b) Acacia saligna, (c) Erophaca baetica, (d) Retama monosperma, (e) Stauracanthus genistoides and (f) Ulex jussiaei. Identification results based on ITS rRNA sequencing. Fig. S3. Non‐metric Multi‐Dimensional Scaling (NMDS) based on culturable and classified genera of endophyte communities isolated from seeds of native and invasive plant species in Dune and Forest (stress value of 0.05). Ellipses represent the two habitats (Dune in yellow or Forest in green) and coloured squares and circles represent plant species within habitats [Invasive species: Acacia melanoxylon (Amel), Acacia saligna (Asal); Native species: Erophaca baetica (Ebae), Genista triacanthos (Gtri), Retama monosperma (Rmon), Stauracanthus genistoides (Sgen) and Ulex jussiaei (Ujus)]. Relative abundance of OTUs in each species was considered for ordination. Table S1. BLAST analysis of sequences obtained from seed bacterial isolation from each plant species. The description shown is for higher percentage pairwise identity. Table S2. BLAST analysis of sequences from fungal isolates from seeds of each plant species. The description shown is for higher percentage pairwise identity. Table S3. Predicted functions for bacterial operational taxonomic units (OTUs), including number of OTU present in each plant species. Functions were predicted through bioinformatic tools using FAPROTAX v. 1.2.6 (Louca et al. 2016). Table S4. Predicted functions for fungal operational taxonomic units (OTUs), including number of OTU present in each plant species. Functions were predicted through bioinformatic tools using FungalTraits (Põl [file PLB-28-249-s001.docx]

**Supplementary Data**

1. ***Acacia melanoxylon***


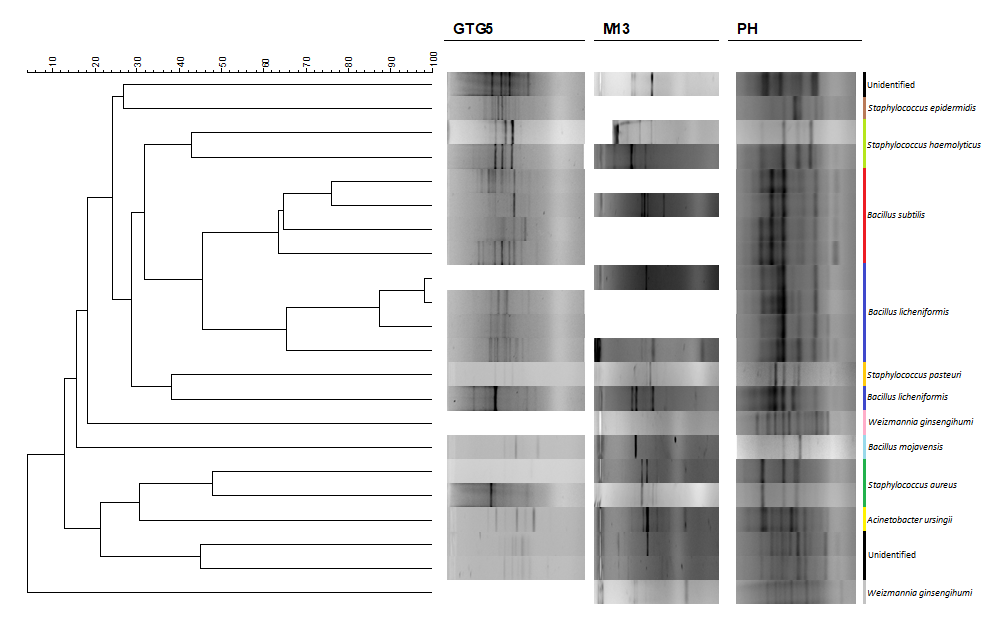


1. ***Acacia saligna***


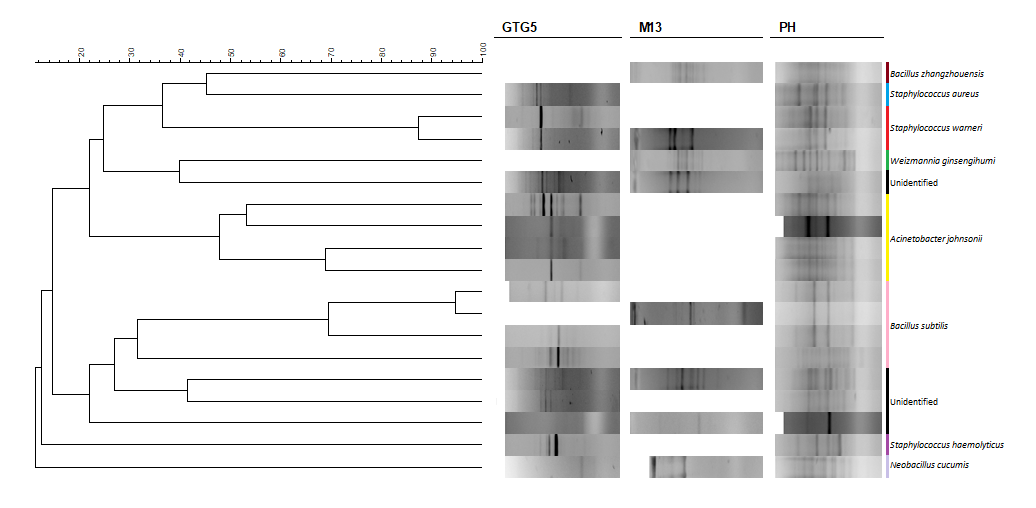


1. ***Erophaca baetica***


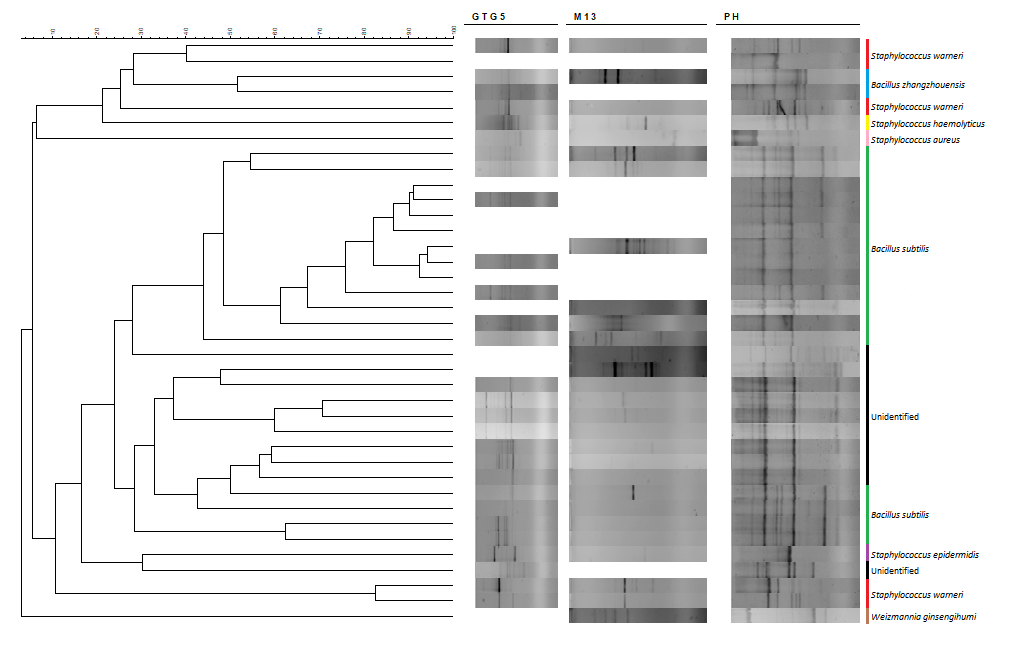


1. ***Retama monosperma***


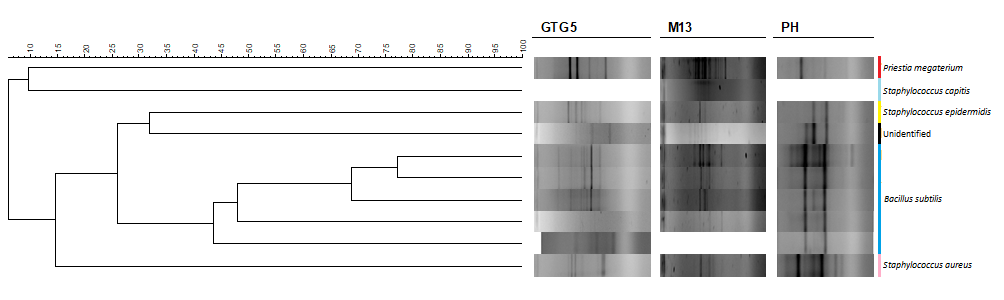


1. ***Stauracanthus genistoides***


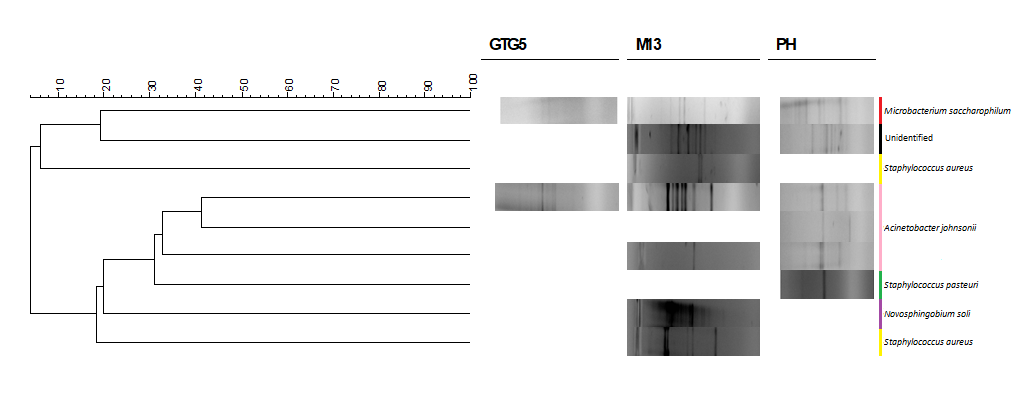


1. ***Ulex jussiaei***


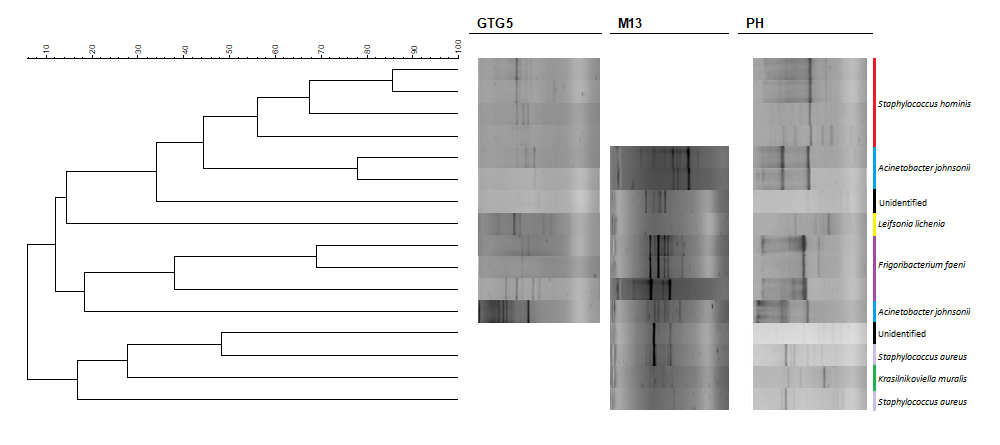


**Supplementary figure 1** Dendrograms of bacterial isolates for each plant species based on DNA fingerprinting: a) *Acacia* melanoxylon, b) Acacia saligna, c) Erophaca baetica, d) Retama monosperma, e) Stauracanthus genistoides and f) Ulex jussiaei. Identification results based on 16S rRNA sequencing are also shown.

1. ***Acacia melanoxylon***


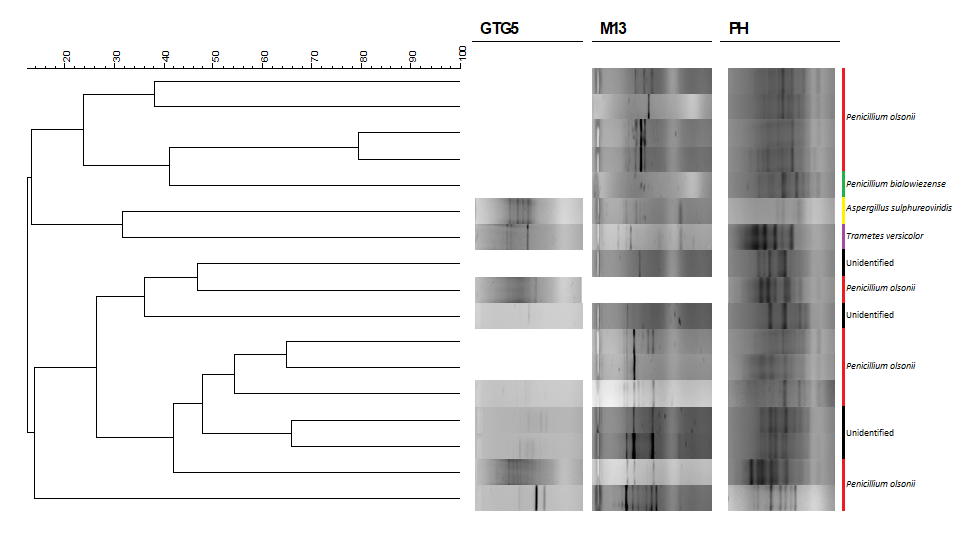


1. ***Acacia saligna***


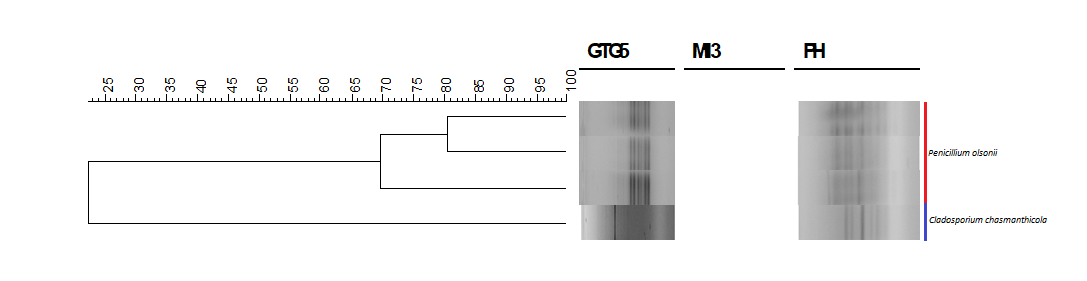


1. ***Erophaca* baetica**


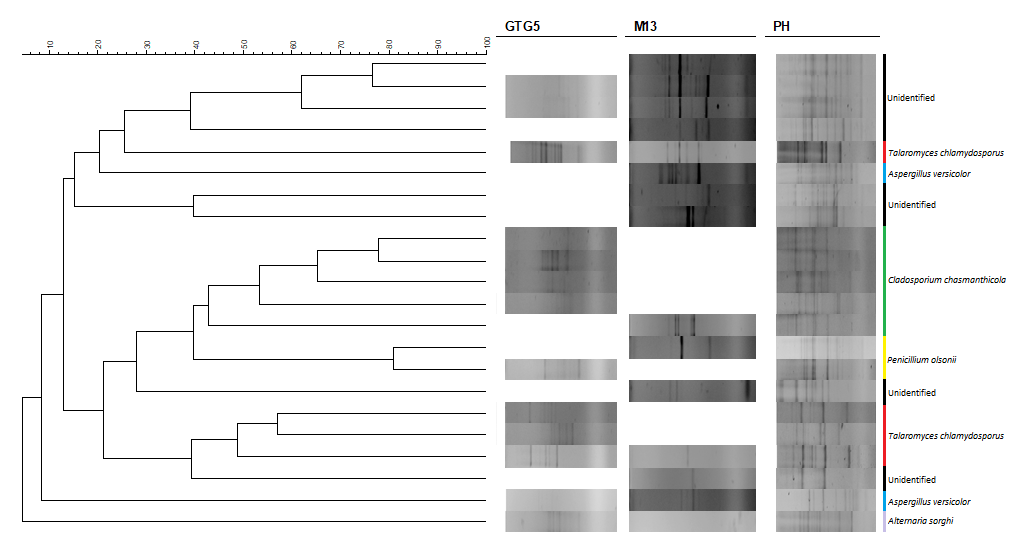


1. ***Retama monosperma***


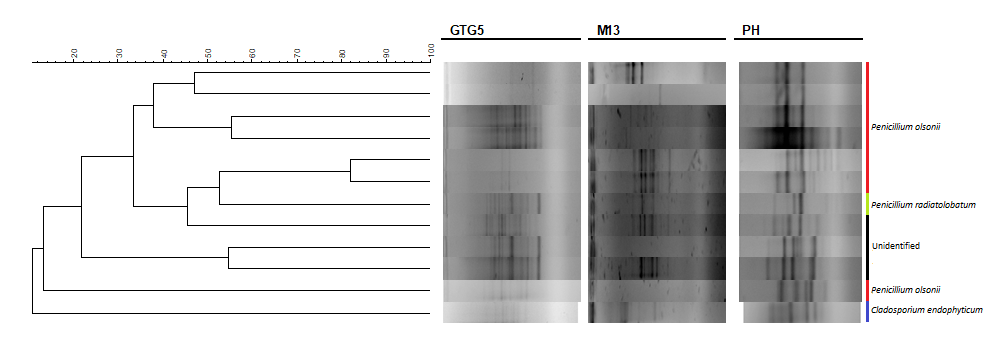


1. ***Stauracanthus genistoides***


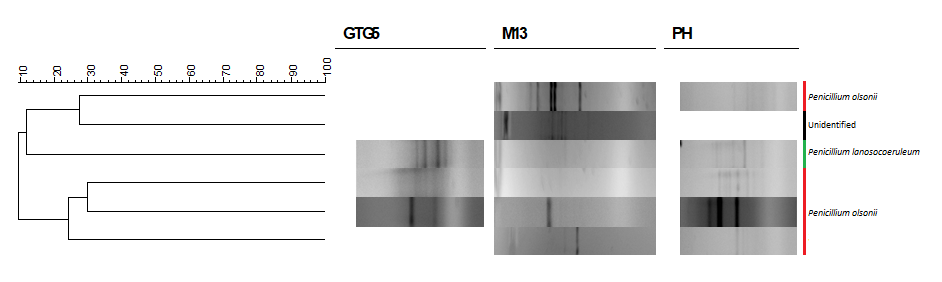


1. ***Ulex jussiaei***


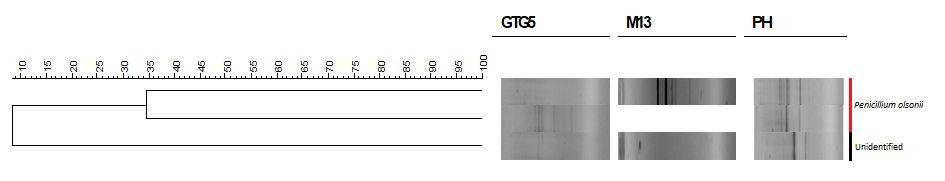


**Supplementary figure 2** Dendrograms of fungal isolates for each plant species based on DNA fingerprinting: a) *Acacia* melanoxylon, b) Acacia saligna, c) Erophaca baetica, d) Retama monosperma, e) Stauracanthus genistoides and f) Ulex jussiaei. Identification results based on ITS rRNA sequencing are also shown.

**Supplementary table** **1** BLAST analysis of the sequences obtained from bacterial isolation from each plant species’ seeds. The description showed is the one that present the higher percentage of pairwise identity. The table continues in the following pages. ^1^Isolates identified as *Weizmannia* spp. were reclassified as *Heyndrickxia* spp. according to the novel classification (Narsing-Rao *et al.*, 2023).

| **Plant species** | **Isolate ID** | **Description** | **% Pairwise**  **Identity** | **GenBank Access Number** |
| --- | --- | --- | --- | --- |
| *Acacia melanoxylon* | AMEL02 | *Leifsonia lichenia* strain 2Sb | 99.7 % | PP703184 |
|  | AMEL03 | *Heyndrickxia ginsengihumi* strain Gsoil 114 ^1^ | 100 % | PP703185 |
|  | AMEL14 | *Acinetobacter ursingii* strain LUH3792 | 100 % | PP703186 |
|  | AMEL16 | *Staphylococcus aureus* strain  ATCC 12600 | 100 % | PP703187 |
|  | AMEL18 | *Bacillus mojavensis* strain IFO 15718 | 100 % | PP703188 |
|  | AMEL20 | *Staphylococcus pasteuri* strain  ATCC 51129 | 99.5 % | PP703189 |
|  | AMEL21 | *Staphylococcus haemolyticus* strain SM 131 | 99.8 % | PP703190 |
|  | AMEL28 | *Staphylococcus epidermidis* strain Fussel | 99.8 % | PP703191 |
|  | AMEL29 | *Bacillus subtilis* strain BCRC 10255 | 100 % | PP703192 |
|  | AMEL33 | *Bacillus licheniformis* strain DSM 13 | 99.9 % | PP703193 |
| *Acacia saligna* | ASAL01 | *Neobacillus cucumis* strain AP-6 | 99.9 % | PP703194 |
|  | ASAL02 | *Bacillus zhangzhouensis* strain MCCC 1A08372 | 100 % | PP703195 |
|  | ASAL03 | *Heyndrickxia ginsengihumi* strain Gsoil 114 ^1^ | 99.8 % | PP703196 |
|  | ASAL04 | *Acinetobacter johnsonii* strain  ATCC 17909 | 99.7 % | PP703197 |
|  | ASAL06 | *Acinetobacter johnsonii* strain  ATCC 17909 | 100 % | PP703198 |
|  | ASAL07 | *Staphylococcus warneri* strain AW 25 | 100 % | PP703199 |
|  | ASAL08 | *Staphylococcus haemolyticus* strain JCM 2416 | 99.9 % | PP703200 |
|  | ASAL10 | *Staphylococcus aureus* strain S33 R | 100 % | PP703201 |
|  | ASAL11 | *Bacillus subtilis* strain BCRC 10255 | 99.9 % | PP703202 |
|  | ASAL22 | *Acinetobacter johnsonii* strain  ATCC 17909 | 99.7 % | PP703203 |
| *Erophaca baetica* | ASTRA02 | *Weizmannia ginsengihumi* strain Gsoil 114 ^1^ | 99.7 % | PP703204 |
|  | ASTRA03 | *Bacillus subtilis* strain IAM 12118 | 99.9 % | PP703205 |
|  | ASTRA14 | *Bacillus zhangzhouensis* strain MCCC 1A08372 | 99.9 % | PP703206 |
|  | ASTRA18 | *Staphylococcus aureus* strain  ATCC 12600 | 100 % | PP703207 |
| **Plant species** | **Isolate ID** | **Description** | **% Pairwise**  **Identity** | **GenBank Access Number** |
| *Erophaca baetica* | ASTRA25 | *Bacillus subtilis* strain IAM 12118 | 100 % | PP703208 |
|  | ASTRA28 | *Staphylococcus warneri* strain AW 25 | 99.9 % | PP703209 |
|  | ASTRA29 | *Staphylococcus warneri* strain AW 25 | 99.6 % | PP703210 |
|  | ASTRA33 | *Bacillus subtilis* strain BCRC 10255 | 100 % | PP703211 |
|  | ASTRA35 | *Staphylococcus warneri* strain AW 25 | 99.9 % | PP703212 |
|  | ASTRA36 | *Staphylococcus epidermidis* strain Fussel | 99.8 % | PP703213 |
|  | ASTRA41 | *Staphylococcus haemolyticus* strain SM 131 | 99.9 % | PP703214 |
| *Genista triacanthos* | GEN01 | *Pseudomonas graminis* strain  DSM 11363 | 100 % | PP703215 |
|  | GEN02 | *Pseudomonas graminis* strain  DSM 11363 | 100 % | PP703216 |
|  | GEN03 | *Pseudomonas graminis* strain  DSM 11363 | 100 % | PP703217 |
|  | GEN07 | *Sphingobium yanoikuyae* strain NBRC 15102 | 99.9 % | PP703218 |
|  | GEN09 | *Sphingobium yanoikuyae* strain  GIFU 9882 | 99.9 % | PP703219 |
|  | GENI02 | *Staphylococcus caprae* strain  ATCC 35538 | 99.9 % | PP703220 |
| *Retama monosperma* | RETAM05 | *Priestia megaterium* strain  ATCC 14581 | 100 % | PP703221 |
|  | RETAM08 | *Bacillus subtilis* strain IAM 12118 | 99.9 % | PP703222 |
|  | RETAM13 | *Staphylococcus aureus* strain  ATCC 12600 | 100 % | PP703223 |
|  | RETAM14 | *Staphylococcus epidermidis* strain Fussel | 99.7 % | PP703224 |
|  | RETAM22 | *Staphylococcus capitis* strain  JCM 2420 | 100 % | PP703225 |
| *Stauracanthus genistoides* | STAUR01 | *Microbacterium saccharophilum* strain K-1 | 99.7 % | PP703226 |
|  | STAUR04 | *Acinetobacter johnsonii* strain  ATCC 17909 | 99.9 % | PP703227 |
|  | STAUR10 | *Staphylococcus aureus* strain S33 R | 100 % | PP703228 |
|  | STAUR11 | *Staphylococcus pasteuri* strain  ATCC 51129 | 100 % | PP703229 |
|  | STAUR13 | *Staphylococcus aureus* strain  ATCC 12600 | 100 % | PP703230 |
|  | STAUR14 | *Staphylococcus capitis* strain  JCM 2420 | 100 % | PP703231 |
|  | STAUR15 | *Novosphingobium soli* strain  CC-TPE-1 | 99.3 % | PP703232 |

| **Plant species** | **Isolate ID** | **Description** | **% Pairwise**  **Identity** | **GenBank Access Number** |
| --- | --- | --- | --- | --- |
| *Ulex jussiaei* | ULEX05 | *Krasilnikoviella muralis* strain  T6220-5-2b | 99.7 % | PP703233 |
|  | ULEX06 | *Acinetobacter johnsonii* strain  ATCC 17909 | 99.7 % | PP703234 |
|  | ULEX08 | *Frigoribacterium faeni* strain 801 | 99.6 % | PP703235 |
|  | ULEX10 | *Leifsonia lichenia* strain 2Sb | 99.7 % | PP703236 |
|  | ULEX13 | *Staphylococcus hominis* strain  DM 122 | 99.8 % | PP703237 |

Narsing Rao, M.P., Banerjee, A., Liu, G.-H., and Thamchaipenet, A. (2023) Genome-based reclassification of *Bacillus acidicola*, *Bacillus pervagus* and the genera *Heyndrickxia*, *Margalitia* and *Weizmannia*. *International Journal of Systematic and Evolutionary Microbiology*, 73,005961. DOI: 10.1099/ijsem.0.005961

**Supplementary table** **2** BLAST analysis of the sequences obtained from fungal isolation from each plant species’ seeds. The description showed is the one that present the higher percentage of pairwise identity.

| **Plant species** | **Isolate ID** | **Description** | **% Pairwise**  **Identity** | **GenBank Access Number** |
| --- | --- | --- | --- | --- |
| *Acacia melanoxylon* | AMEL01 | *Penicillium olsonii* CBS 232.60 | 100 % | PP704364 |
|  | AMEL04 | *Penicillium olsonii* CBS 232.60 | 99.9 % | PP704365 |
|  | AMEL05 | *Penicillium olsonii* CBS 232.60 | 100 % | PP704366 |
|  | AMEL07 | *Penicillium bialowiezense* CBS 227.28 | 99.1 % | PP704367 |
|  | AMEL19 | *Penicillium olsonii* CBS 232.60 | 100 % | PP704368 |
|  | AMEL22 | *Aspergillus sulphureoviridis*  CBS 140626 | 98.7 % | PP704369 |
|  | AMEL25 | *Trametes versicolor*  CFMR FP-135156-Sp | 99.6 % | PP704370 |
|  | AMEL26 | *Penicillium olsonii* CBS 232.60 | 100 % | PP704371 |
|  | AMEL39 | *Penicillium olsonii* CBS 232.60 | 100 % | PP704372 |
| *Acacia saligna* | ASAL12 | *Cladosporium chasmanthicola*  CPC 21300 | 100 % | PP704373 |
|  | ASAL13 | *Penicillium olsonii* CBS 232.60 | 100 % | PP704374 |
| *Erophaca baetica* | ASTRA01 | *Penicillium olsonii* CBS 232.60 | 100 % | PP704375 |
|  | ASTRA04 | *Aspergillus versicolor* ATCC 9577 | 99.1 % | PP704376 |
|  | ASTRA06 | *Aspergillus versicolor* ATCC 9577 | 98.9 % | PP704377 |
|  | ASTRA19 | *Alternaria sorghi* CBS 127502 | 99.9 % | PP704378 |
|  | ASTRA26 | *Talaromyces chlamydosporus*  CBS 140635 | 97.7 % | PP704379 |
|  | ASTRA40 | *Penicillium olsonii* CBS 232.60 | 100 % | PP704380 |
|  | ASTRA43 | *Cladosporium chasmanthicola*  CPC 21300 | 100 % | PP704381 |
| *Genista triacanthos* | GENI04 | *Alternaria alstroemeriae* CBS 118809 | 99.9 % | PP704382 |
|  | GENI05 | *Penicillium olsonii* CBS 232.60 | 100 % | PP704383 |
|  | GENI06 | *Penicillium olsonii* CBS 232.60 | 100 % | PP704384 |
|  | GENI08 | *Penicillium olsonii* CBS 232.60 | 100 % | PP704385 |
|  | RETAM01 | *Penicillium olsonii* CBS 232.60 | 100 % | PP704386 |
|  | RETAM04 | *Penicillium radiatolobatum*  CBS 340.79 | 100 % | PP704387 |
| *Retama monosperma* | RETAM10 | *Penicillium olsonii* CBS 232.60 | 100 % | PP704388 |
|  | RETAM16 | *Penicillium olsonii* CBS 232.60 | 100 % | PP704389 |
|  | RETAM18 | *Penicillium olsonii* CBS 232.60 | 100 % | PP704390 |
|  | RETAM21 | *Cladosporium endophyticum* MFLUCC 17-0599 | 99.6 % | PP704391 |
|  |  |  |  |  |
|  |  |  |  |  |
| **Plant species** | **Isolate ID** | **Description** | **% Pairwise**  **Identity** | **GenBank Access Number** |
| *Stauracanthus genistoides* | STAUR02 | *Penicillium olsonii* CBS 232.60 | 100 % | PP704392 |
|  | STAUR03 | *Penicillium lanosocoeruleum*  CBS 215.30 | 99.7 % | PP704393 |
|  | STAUR05 | *Penicillium olsonii* CBS 232.60 | 100 % | PP704394 |
|  | STAUR07 | *Penicillium olsonii* CBS 232.60 | 100 % | PP704395 |
|  | STAUR12 | *Penicillium olsonii* CBS 232.60 | 100 % | PP704396 |
| *Ulex jussiaei* | ULEX07 | *Penicillium olsonii* CBS 232.60 | 99.9 % | PP704397 |


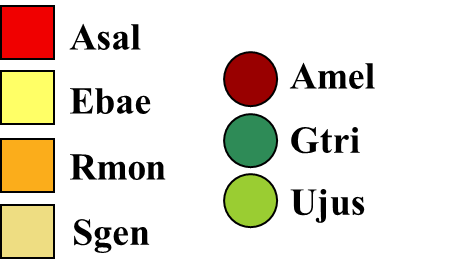

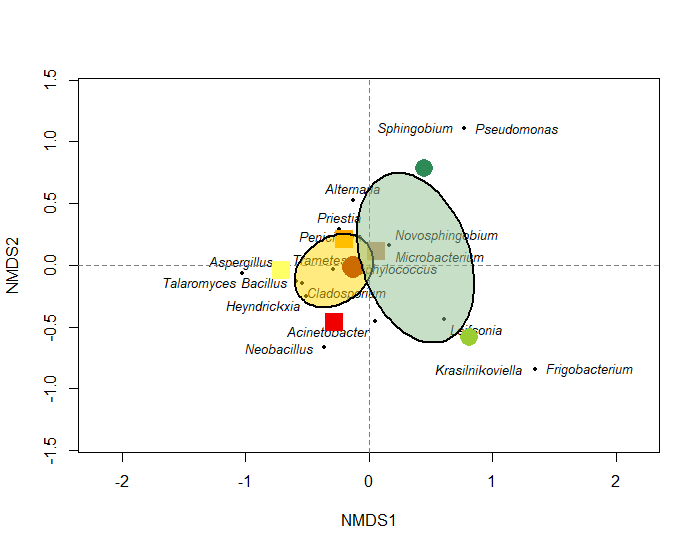


**Supplementary figure 3** Two-dimensional Non-metric MultiDimensional Scaling (NMDS) ordination based on culturable and classified genera of endophytic communities isolated from seeds of native and invasive plant species in Dune and Forest (stress value of 0.05). Ellipses represent the two studied habitats (Dune in yellow or Forest in green) and colored squares and circles represent plant species within habitats [Invasive species: *Acacia melanoxylon* (Amel)*, Acacia saligna* (Asal); Native species: *Erophaca baetica* (Ebae)*, Genista triacanthos* (Gtri)*, Retama monosperma* (Rmon)*, Stauracanthus genistoides* (Sgen) and *Ulex jussiaei* (Ujus)]*.* Relative abundance of OTUs in each plant species was considered for ordination.

**Supplementary table** **3** Predicted functions for bacterial operational taxonomic units (OTUs), including the number of OTU present in each plant species. Functions were predicted through bioinformatic tools using FAPROTAX v1.2.6 (Louca *et al*. 2016).

|  | **Plant species** | | | | | | | **Predicted functions** | | | | | | | | | | | | | | | |
| --- | --- | --- | --- | --- | --- | --- | --- | --- | --- | --- | --- | --- | --- | --- | --- | --- | --- | --- | --- | --- | --- | --- | --- |
| **OTU** | 1. *saligna* | *E. baetica* | *R. monosperma* | *S. genistoides* | *A. melanoxylon* | *G. triacanthos* | *U. jussiaei* | Animal associated | Plant pathogen | N respiration | Nitrate reduction | Nitrate respiration | Nitrite respiration | Nitrate ammonification | Nitrite ammonification | Xylanolysis | Ligninolysis | Urelolysis | Cellulolysis | Chemoheterotrophy | Chitinolysis | Fermentation | Aromatic compound degradation |
| *Acinetobacter johnsonii* | 4 |  |  | 3 |  |  | 3 |  |  |  |  |  |  |  |  |  |  |  |  | x |  |  | x |
| *Acinetobacter ursingii* |  |  |  |  | 1 |  |  |  |  |  |  |  |  |  |  |  |  |  |  | x |  |  | x |
| *Bacillus licheniformis* |  |  |  |  | 5 |  |  |  |  |  | x |  |  |  |  |  |  |  |  | x | x | x |  |
| *Bacillus mojavensis* |  |  |  |  | 1 |  |  |  |  |  | x |  |  |  |  |  |  |  |  |  |  |  |  |
| *Bacillus subtilis* | 4 | 17 | 5 |  | 4 |  |  |  |  | x | x | x | x | x | x | x | x |  | x | x |  |  |  |
| *Bacillus zhangzhouensis* | 1 | 2 |  |  |  |  |  |  |  |  |  |  |  |  |  |  |  |  |  |  |  |  |  |
| *Frigoribacterium faeni* |  |  |  |  |  |  | 3 |  |  |  |  |  |  |  |  |  |  |  |  | x |  |  |  |
| *Heyndrickxia ginsengihumi* | 1 | 1 |  |  | 1 |  |  |  |  |  |  |  |  |  |  |  |  |  |  |  |  |  |  |
| *Krasilnikoviella muralis* |  |  |  |  |  |  | 1 |  |  |  |  |  |  |  |  |  |  |  |  |  |  |  |  |
| *Leifsonia lichenia* |  |  |  |  | 1 |  | 1 |  |  |  |  |  |  |  |  |  |  |  |  | x |  |  |  |
| *Microbacterium saccharophilum* |  |  |  | 1 |  |  |  |  |  |  |  |  |  |  |  |  |  |  |  | x |  |  |  |
| *Neobacillus cucumis* | 1 |  |  |  |  |  |  |  |  |  |  |  |  |  |  |  |  |  |  |  |  |  |  |
| *Novosphingobium soli* |  |  |  | 1 |  |  |  |  |  |  |  |  |  |  |  |  |  |  |  | x |  |  |  |
| *Priestia megaterium* |  |  | 1 |  |  |  |  |  |  |  |  |  |  |  |  |  |  |  |  |  |  |  |  |
| *Pseudomonas graminis* |  |  |  |  |  | 3 |  |  |  |  |  |  |  |  |  |  |  |  |  | x |  |  |  |
| *Staphylococcus aureus* | 1 | 1 | 1 | 2 | 2 |  |  | x | x |  | x |  |  |  |  |  |  |  |  |  |  |  |  |
| *Staphylococcus capitis* |  |  | 1 | 1 |  |  |  | x |  |  | x |  |  |  |  |  |  |  |  |  |  |  |  |
| *Staphylococcus epidermidis* |  |  | 1 |  | 1 |  |  | x |  |  | x |  |  |  |  |  |  | x |  |  |  |  |  |
| *Staphylococcus haemolyticus* | 1 | 1 |  |  | 2 |  |  |  |  |  |  |  |  |  |  |  |  |  |  |  |  |  |  |
| *Staphylococcus hominis* |  |  |  |  |  |  | 4 |  |  |  |  |  |  |  |  |  |  | x |  |  |  |  |  |
| *Staphylococcus pasteuri* |  |  |  | 1 | 1 |  |  |  |  |  |  |  |  |  |  |  |  | x |  |  |  |  |  |
| *Staphylococcus warneri* | 2 | 5 |  |  |  |  | 2 | x |  |  |  |  |  |  |  |  |  | x |  |  |  |  |  |
| *Sphingobium yanoikuyae* |  |  |  |  |  | 1 |  |  |  |  |  |  |  |  |  |  |  |  |  | x |  |  |  |

**Supplementary table** **4** Predicted functions for fungal operational taxonomic units (OTUs), including the number of OTU present in each plant species. Functions were predicted through bioinformatic tools using FungalTraits (Põlme *et al*., 2020).

|  | **Plant species** | | | | | | | **Predicted functions** | | | | | | | | | | **Traits** | | | | | | |  |  |
| --- | --- | --- | --- | --- | --- | --- | --- | --- | --- | --- | --- | --- | --- | --- | --- | --- | --- | --- | --- | --- | --- | --- | --- | --- | --- | --- |
| **OTU** | 1. *saligna* | *E. baetica* | *R. monosperma* | *S. genistoides* | *A. melanoxylon* | *G. triacanthos* | *U. jussiaei* | Animal parasite | Plant pathogen | Plant endophyte | Decay type: soft rot | Decay type: white rot | Decay: mold | Decay: plant material | Decay: animal material | Decay: soil | Aquatic | | With hymenium | Filamentous | Polyporoid | Primary life: plant pathogen | Primary life: saprotroph | Second life: foliar endophyte | | Second life: plant pathogen |
| *Alternaria alstroemeriae* |  |  |  |  |  | 1 |  |  | x | x | x |  |  | x |  |  | x | | x | x |  | x | x |  | |  |
| *Alternaria sorghi* |  | 1 |  |  |  |  |  |  | x | x | x |  |  | x |  |  | x | | x | x |  | x | x |  | |  |
| *Aspergillus sulphureoviridis* |  |  |  |  | 1 |  |  | x |  | x |  |  | x | x | x | x | x | |  | x |  |  | x | x | |  |
| *Aspergillus versicolor* |  | 2 |  |  |  |  |  | x |  | x |  |  | x | x | x | x | x | |  | x |  |  |  | x | |  |
| *Cladosporium chasmanthicola* | 1 | 5 |  |  |  |  |  | x | x | x |  |  |  | x | x | x | x | | x | x |  |  | x |  | | x |
| *Cladosporium endophyticum* |  |  | 1 |  |  |  |  | x | x |  |  |  |  | x | x | x | x | | x | x |  |  | x |  | | x |
| *Penicillium bialowiezense* |  |  |  |  | 1 |  |  | x |  | x |  |  | x |  |  | x | x | |  | x |  |  | x | x | |  |
| *Penicillium lanosocoeruleum* |  |  |  | 1 |  |  |  | x |  | x |  |  | x |  |  | x | x | |  | x |  |  | x | x | |  |
| *Penicillium olsonii* | 3 | 2 | 7 | 4 | 10 | 3 | 2 | x |  | x |  |  | x |  |  | x | x | |  | x |  |  | x | x | |  |
| *Penicillium radiatolobatum* |  |  | 1 |  |  |  |  | x |  | x |  |  | x |  |  | x | x | |  | x |  |  | x | x | |  |
| *Talaromyces chlamydosporus* |  | 4 |  |  |  |  |  | x |  |  |  |  | x | x | x | x | x | |  | x |  |  |  |  | |  |
| *Trametes versicolor* |  |  |  |  | 1 |  |  |  |  |  |  | x |  | x |  |  | x | | x | x | x |  | x |  | |  |
